# Adjacency-constrained hierarchical clustering of a band similarity matrix with application to genomics

**DOI:** 10.1186/s13015-019-0157-4

**Published:** 2019-11-15

**Authors:** Christophe Ambroise, Alia Dehman, Pierre Neuvial, Guillem Rigaill, Nathalie Vialaneix

**Affiliations:** 1 Laboratoire de Mathématiques et Modélisation d’Evry, UMR CNRS 8071, Université d’Evry Val d’Essonne, 23 boulevard de France, 91037 Evry, France; 2 Hyphen-stat, 195 Route d’Espagne, 31036 Toulouse, France; 3 Institut de Mathématiques de Toulouse, UMR5219 CNRS, Université de Toulouse, UPS IMT, 31062 Toulouse Cedex 9, France; 4 Institute of Plant Sciences Paris Saclay IPS2, CNRS, INRA, Gif sur Yvette, France; 5 MIAT, Université de Toulouse, INRA, Castanet-Tolosan, France

**Keywords:** Hierarchical agglomerative clustering, Adjacency constraint, Segmentation, Ward’s linkage, Similarity, Min heap, Genome-Wide Association Studies and Hi-C

## Abstract

**Background:**

Genomic data analyses such as Genome-Wide Association Studies (GWAS) or Hi-C studies are often faced with the problem of partitioning chromosomes into successive regions based on a similarity matrix of high-resolution, locus-level measurements. An intuitive way of doing this is to perform a modified Hierarchical Agglomerative Clustering (HAC), where only adjacent clusters (according to the ordering of positions within a chromosome) are allowed to be merged. But a major practical drawback of this method is its quadratic time and space complexity in the number of loci, which is typically of the order of $10^4$ to $10^5$ for each chromosome.

**Results:**

By assuming that the similarity between physically distant objects is negligible, we are able to propose an implementation of adjacency-constrained HAC with quasi-linear complexity. This is achieved by pre-calculating specific sums of similarities, and storing candidate fusions in a min-heap. Our illustrations on GWAS and Hi-C datasets demonstrate the relevance of this assumption, and show that this method highlights biologically meaningful signals. Thanks to its small time and memory footprint, the method can be run on a standard laptop in minutes or even seconds.

**Availability and implementation:**

Software and sample data are available as an R package, **adjclust**, that can be downloaded from the Comprehensive R Archive Network (CRAN).

## Background

Genetic information is coded in long strings of DNA organised in chromosomes. High-throughput sequencing such as RNAseq, DNAseq, ChipSeq and Hi-C makes it possible to study biological phenomena along the entire genome at a very high resolution [32].

In most cases, we expect neighboring positions to be statistically dependent. Using this *a priori* information is one way of addressing the complexity of genome-wide analyses. For instance, it is common practice to partition each chromosome into regions, because such regions hopefully correspond to biological relevant or interpretable units (such as genes or binding sites) and because statistical modelling and inference are simplified at the scale of an individual region. In simple cases, such regions are given (for example, in RNAseq analysis, only genic and intergenic regions are usually considered and differential analysis is commonly performed at the gene or transcript level). However, in more complex cases, regions of interest are unknown and need to be discovered by mining the data. This is the case in the two leading examples considered in this paper. In the context of Genome Wide Association Studies (GWAS), region-scale approaches taking haplotype blocks into account can result in substantial statistical gains [17]. Hi-C studies [12] have demonstrated the existence of topological domains, which are megabase-sized local chromatin interaction domains correlating with regions of the genome that constrain the spread of heterochromatin. Hence, the problem of partitioning a chromosome into biologically relevant regions based on measures of similarity between pairs of individual loci has been extensively studied for genomic applications.

Recovering the “best” partition of *p* loci for each possible number, *K*, of classes is equivalent to a segmentation problem (also known as “multiple changepoint problem”). In the simplest scenario where the signals to be segmented are piecewise-constant, such as in the case of DNA copy numbers in cancer studies, segmentation can be cast as a least squares minimization problem [23, 30]. More generally, kernel-based segmentation methods have been developed to perform segmentation on data described by a similarity measure [3, 22]. Such segmentation problems are combinatorial in nature, as the number of possible segmentations of *p* loci into *K* blocks (for a given $K=1 \dots p$) is ${p \atopwithdelims ()K} = \mathcal {O}(p^K)$. The “best” segmentation for all $K=1 \dots p$ can be recovered efficiently in a quadratic time and space complexity using dynamic programming. As discussed in Celisse et al. [7], in the case of kernel-based segmentation, this complexity cannot be improved without making additional assumptions on the kernel (or the corresponding similarity). Indeed, for a generic kernel, even computing the loss (that is, the least square error) of any given segmentation in a fixed number of segments *K* has a computational cost of $\mathcal {O}(p^2)$.

The goal of this paper is to develop heuristics that can be applied to genomic studies in which the number of loci is so large (typically of the order of $p=10^4$ to $10^6$) that algorithms of quadratic time and space complexity cannot be applied. This paper stems from a modification of the classical hierarchical agglomerative clustering (HAC) [26], where only adjacent clusters are allowed to be merged. This simple constraint is well-suited to genomic applications, in which loci can be ordered along chromosomes provided that an assembled genome is available. Adjacency-constrained HAC can be seen as a heuristic for segmentation; it provides not only a single partition of the original loci, but a sequence of nested partitions.

The idea of incorporating such constraints was previously mentioned by Lebart [27] to incorporate geographical (two-dimensional) constraints to cluster socio-economic data, and by  Michel et al. [28] to cluster functional Magnetic Resonance Imaging (fMRI) data into contiguous (three-dimensional) brain regions. The totally ordered case that is the focus of this paper has been studied by Grimm [19], and an R package implementing this algorithm, **rioja** [25], has been developed.^1^ However, the algorithm remains quadratic in both time and space. Its time complexity cannot be improved because all of the $p^2$ similarities are used in the course of the algorithm. To circumvent this difficulty, we assume that the similarity between physically distant loci is zero, where two loci are deemed to be “physically distant” if they are separated by more than *h* other loci. The main contribution of this paper is to propose an adjacency-constrained clustering algorithm with quasi-linear complexity [namely, $\mathcal {O}(ph)$ in space and $\mathcal {O}(p(h + \log (p)))$ in time] under this assumption, and to demonstrate its relevance for genomic studies. This algorithm is obtained by combining (i) constant-time calculation of Ward’s likage after a pre-calculation step of linear time and space complexity, and (ii) storage of candidate fusions in a binary heap.

The rest of the paper is organized as follows. In “Method” section we describe the algorithm, its time and space complexity and its implementation. The resulting segmentation method is then applied to GWAS datasets (“Linkage disequilibrium block inference in GWAS” section) and to Hi-C datasets (“Hi-C analysis” section), in order to illustrate that the above assumption makes sense in such studies, and that the proposed methods can be used to recover biologically relevant signals.

## Method

### Adjacency-constrained HAC with Ward’s linkage

In its unconstrained version, HAC starts with a trivial clustering where each object is in its own cluster and iteratively merges the two most similar clusters according to a distance function $\delta$ called a linkage criterion. We focus on Ward’s linkage, which was defined for clustering objects $(x_i)_i$ taking values in the Euclidean space $\mathbb {R}^d$. Formally, Ward’s linkage between two clusters *C* and $C'$ defines the distance between two clusters as the increase in the error sum of squares (or equivalently, as the decrease in variance) when *C* and $C'$ are merged: $\delta (C,C') = \text{ ESS }(C \cup C') - \text{ ESS }(C) - \text{ ESS }(C')$, where $\text{ ESS }(C) := \frac{1}{|C|} \sum _{i \in C} \Vert x_i - \bar{C}\Vert ^2_{\mathbb {R}^d}$ is the Error Sum of Squares of cluster *C* (also known as “inertia of *C*”) and $\bar{C} = \frac{1}{n} \sum _{i \in C} x_i$. It is one of the most widely used linkages because of its natural interpretation in terms of within/between cluster variance and because HAC with Ward’s linkage can be seen as a greedy algorithm for least square minimization, similarly to the *k*-means algorithm. In this paper, the *p* objects to be clustered are assumed to be ordered by their indices $i \in \left\{ 1, \dots p\right\}$. We focus on a modification of HAC where only adjacent clusters are allowed to be merged. This *adjacency-constrained* HAC is described in Algorithm 1.

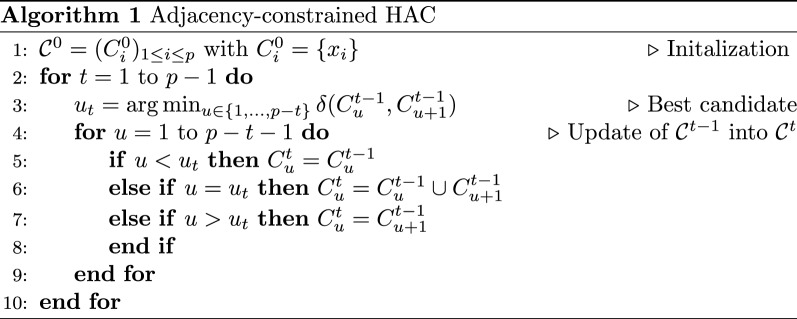



An implementation in Fortran of this algorithm was provided by Grimm [19]. This implementation has been integrated in the R package rioja [25].

### Extension to general similarities

HAC and adjacency-constrained HAC are frequently used when the objects to be clustered do not belong to $\mathbb {R}^d$ but are described by pairwise dissimilarities that are not necessarily Eulidean distance matrices. This case has been formally studied in Székely and Rizzo [35], Strauss and von Maltitz [34], Chavent et al. [8] and generally involves extending the linkage formula by making an analogy between the dissimilarity and the distance in $\mathbb {R}^d$ (or the squared distance in some cases). These authors have shown that the simplified update of the linkage at each step of the algorithm, known as the Lance-Williams formula, is still valid in this case and that the objective criterion can be interpreted as the minimization of a so-called “pseudo inertia”. A similar approach can be used to extend HAC to data described by an arbitrary similarity between objects, $S = (s_{ij})_{i,j=1,\ldots ,p}$, using a kernel framework as in [1, 31]. More precisely, when *S* is positive definite, the theory of Reproducing Kernel Hilbert Spaces [4] implies that the data can be embedded in an implicit Hilbert space. This allows to formulate Ward’s linkage between any two clusters in terms of the similarity using the so-called “kernel trick”: $\forall \, C,\ C' \subset \{1,\ldots ,p\}$,

1 $$\begin{aligned} \delta (C,C') = \frac{S(C)}{|C|} + \frac{S(C')}{|C'|} - \frac{ S(C \cup C')}{|C \cup C'|}\,, \end{aligned}$$

where $S(C) = \sum _{(i,j) \in C^2} s_{ij}$ only depends on *S* and not on the embedding. This expression shows that Ward’s Linkage also has a natural interpretation as the decrease in average intra-cluster similarity after merging two clusters. Equation (1) is proved in Section S1.1 of Additional file 1.

Extending this approach to the case of a general (that is, possibly non-positive definite) similarity matrix has been studied in Miyamoto et al. [29]. Noting that (*i*) for a large enough $\lambda$, the matrix $S_\lambda = S+\lambda I_p$ is positive definite and that (*ii*) $\delta _{S_\lambda }(C,C') = \delta (C,C') + \lambda$, Miyamoto et al. [29, Theorem 1] concluded that applying Ward’s HAC to *S* and $S_\lambda$ yields the exact same hierarchy, only shifting the linkage values by $+\lambda$. This result, which a fortiori holds for the adjacency-constrained Ward’s HAC, justifies the use of Eq. (1) in the case of a general similarity matrix.

### Band similarity assumption

In the case described in “Adjacency-constrained HAC with Ward’s linkage” section where the *p* objects to be clustered belong to $\mathbb {R}^d$, with $d<p$, the computation of Ward’s linkage between two clusters can be done in $\mathcal {O}(d)$ by exploiting its explicit alternative formulation as the distance between centers of gravity. In such cases, it is possible to obtain unconstrained HAC in $\mathcal {O}(p^2 \log ^2 p)$ in time [14], and lower complexities could possibly be achieved for adjacency-constrained HAC. However, we focus in this paper in the situation described in “Extension to general similarities” section, where the input objects are represented by pairwise similarities. In such cases there is generally no explicit or finite-dimensional representation of the centers of gravity, and the time complexity of adjacency-constrained HAC (e.g. in **rioja**) is intrinsically quadratic in *p* because all of the $p^2$ similarities are used to compute all of the required linkage values (Algorithm 1, line 3).

Note that the implementation provided in **rioja** is also quadratic in space, as it takes as an input a $p \times p$ (dense) dissimilarity matrix. However, Algorithm 1 can be made sub-quadratic in space in situations where the similarity matrix is sparse (see Ah-Pine and Wang [1] for similar considerations in the unconstrained case) or when the similarities can be computed on the fly, that is, at the time they are required by the algorithm, as in Dehman et al. [11].

In applications where adjacency-constrained clustering is relevant, such as Hi-C and GWAS data analysis, this quadratic time complexity is a major practical bottleneck because *p* is typically of the order of $10^4$ to $10^5$ for each chromosome. Fortunately, in such applications it also makes sense to assume that the similarity between physically distant objects is small. Specifically, we assume that *S* is a band matrix of bandwidth $h+1$, where $h \in \{1\dots p\}$: $s_{ij} = 0$ for $|i-j| \ge h$. This assumption is not restrictive, as it is always fulfilled for $h=p$. However, we will be mostly interested in the case where $h \ll p$. In the next section, we introduce an algorithm with improved time and space complexity under this band similarity assumption.

## Algorithm

### Ingredients

Our proposed algorithm relies on (*i*) constant-time calculation of each of the Ward’s linkages involved at line 3 of Algorithm 1 using Eq. (1), and (*ii*) storage of the candidate fusions in a min-heap. These elements are described in the next two subsections.

#### Ward’s linkage as a function of pre-calculated sums

**Fig. 1:**
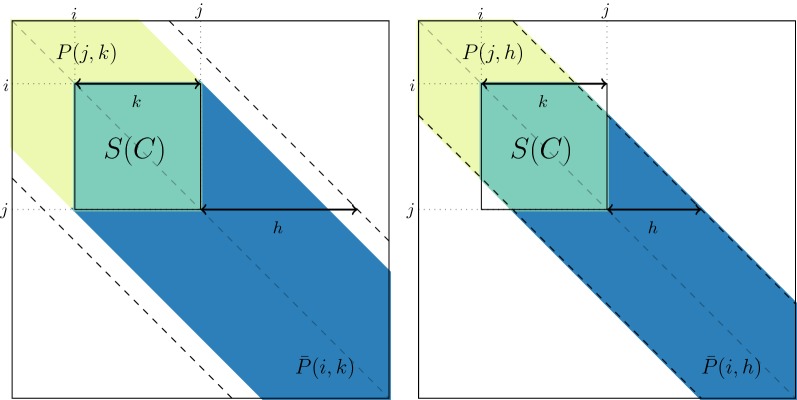
Example of forward pencils (in yellow and green) and backward pencils (in green and blue), and illustration of Eq. (3) for cluster $C=\{i, \ldots , j-1\}$. Left: cluster smaller than bandwidth ($k \le h$); right: cluster larger than bandwidth $k \ge h$

The key point of this subsection is to show that the sums of similarities involved in Eq. (1) may be expressed as a function of certain pre-calculated sums. We start by noting that the sum of all similarities in any cluster $C=\{i, \dots , j-1\}$ of size $k=j-i$ can easily be obtained from sums of elements in the first $\min (h,k)$ subdiagonals of *S*. To demonstrate that this is the case we define, for $1 \le r,l \le p$, *P*(*r*, *l*) as the sum of all elements of *S* in the first *l* subdiagonals of the upper-left $r \times r$ block of *S*. Formally,

2 $$\begin{aligned} P(r,l) = \sum _{1\le i,j \le r, |i-j| < l} s_{ij} \end{aligned}$$

and symmetrically, $\bar{P}(r, l) = P(p+1-r, l)$. This notation is illustrated in Fig. 1, with $r \in \{i,j\}$. In the left panel, $l=k \le h$, while in the right panel, $l=h \le k$. In both panels, $P(j, \min (h,k))$ is the sum of elements in the yellow and green regions, while $\bar{P}(i, \min (h,k))$ is the sum of elements in the green and blue regions. Because *P* and $\bar{P}$ are sums of elements in pencil-shaped areas, we call *P*(*r*, *l*) a *forward pencil* and $\bar{P}(r,l)$ a *backward pencil*.

Figure 1 illustrates that the sum $S_{CC}$ of all similarities in cluster *C* can be computed from forward and backward pencils using the identity:

3 $$\begin{aligned} P(j, h_k) + \bar{P}(i, h_k) = S(C) + P(p, h_k), \end{aligned}$$

where $h_k:=\min (h,k)$ and $P(p, h_k)$ is the “full” pencil of bandwidth $h_k$ (which also corresponds to $\bar{P}(1, h_k)$). The above formula makes it possible to compute $\delta (C,C')$ in constant time from the pencil sums using Eq. (1). By construction, all the bandwidths of the pencils involved are less than *h*. Therefore, only pencils *P*(*r*, *l*) and $\bar{P}(r,l)$ with $1 \le r \le p$ and $1 \le l \le h$ have to be pre-computed, so that the total number of pencils to compute and store is less than 2*ph*. These computations can be performed recursively in a $\mathcal {O}(ph)$ time complexity. Further details about the time and space complexity of this pencil trick are given in Section S1.2 of Additional file 1.

#### Storing candidate fusions in a min-heap

**Fig. 2:**
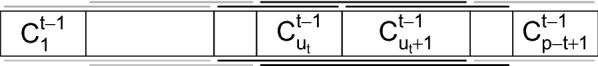
The $t^{\rm th}$ merging step in adjacency-constrained HAC in Algorithm 1. The clusters are represented by rectangular cells. Candidate fusions are represented by horizontal bars: above the corresponding pair of clusters at step *t* and below it at step $t+1$, assuming that the best fusion is the one between the clusters of indices $u_t$ and $u_t+1$. Gray bars indicate candidate fusions that are present at both steps

Iteration *t* of Algorithm 1 consists in finding the minimum of $p-t$ elements, corresponding to the candidate fusions between the $p-t+1$ clusters in ${\mathcal {C}}^{t-1}$, and merging the corresponding clusters. Storing the candidate fusions in an *unordered array* and calculating the minimum at each step would mean a quadratic time complexity. One intuitive strategy would be to make use of the fact that all but 2 to 3 candidate fusions at step *t* are still candidate fusions at step $t-1$, as illustrated by Fig. 2 where candidate fusions are represented by horizontal bars above the clusters. However, maintaining a *totally-ordered* list of candidate fusions is not efficient because the cost of deleting and inserting an element in an ordered list is linear in *p*, again leading to a quadratic time complexity. Instead, we propose storing the candidate fusions in a *partially-ordered* data structure called a *min heap*  [36]. This type of structure achieves an appropriate tradeoff between the cost of maintaining the structure and the cost of finding the minimum element at each iteration, as illustrated in Table 1.

**Table 1:** Time complexities ($\times \mathcal {O}(1)$) of the three main elementary operations required by one step of adjacency-constrained clustering (in columns), for three implementation options (in rows), for a problem of size *p*

	Find $\min$	Insert	Delete $\min$	Total
Unordered array	*p*	1	*p*	*p*
Min heap	1	$\log (p)$	$\log (p)$	$\log (p)$
Ordered array	1	*p*	*p*	*p*

A min heap is a binary tree such that the value of each node is smaller than the value of its two children. The advantage of this structure is that all the operations required in Algorithm 1 to create and maintain the list of candidate fusions can be done very efficiently. We provide a detailed description of the method, which is implemented in the **adjclust** package. We also give illustrations of the first steps of this algorithm when applied to the RLGH data set provided in the package **rioja**, that are relative abundances of 41 taxa in $p=20$ stratigraphic samples. A detailed description of this data set is provided in the help of the RLGH data set.

### Proposed algorithm

#### Description and illustration

Our proposed algorithm is summarized by Algorithm 2. It is best expressed in terms of candidate fusions, contrary to Algorithm 1 which was naturally described in terms of clusters.

**Fig. 3:**
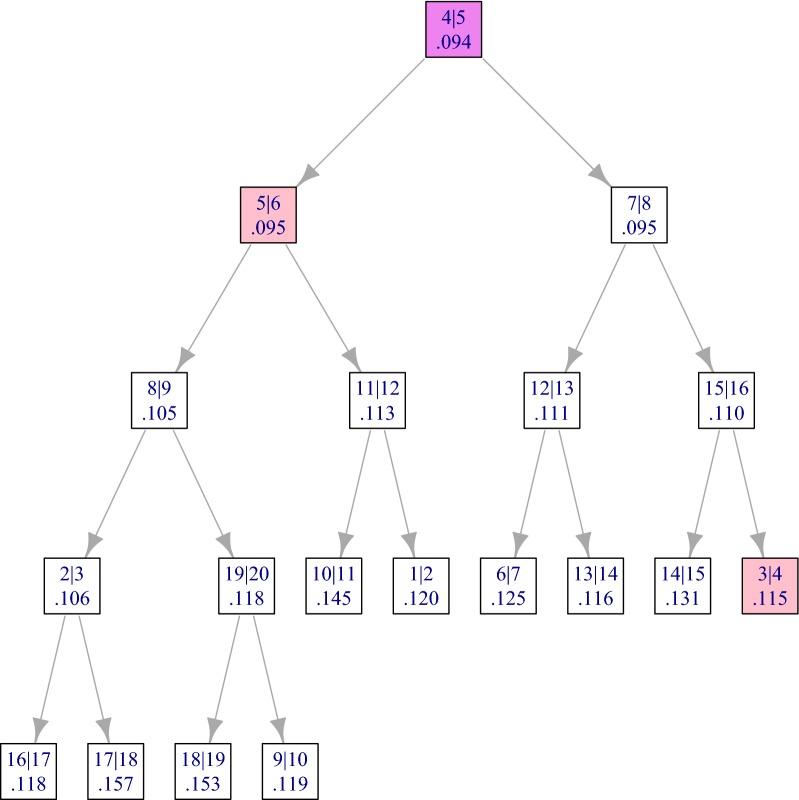
Min heap after the initialization step of the RLGH data set. Each node corresponds to a candidate fusion, and is represented by a label of the form $i \vert i+1$ giving the indices of the items to be merged, and (ii) the value of the corresponding linkage $\delta (\left\{ i\right\} , \left\{ j\right\} )$. The nodes corresponding to the best fusion and the two neighbor fusions are highlighted

The initialization step (lines 1 to 3) consists in building the heap of $p-1$ candidate fusions between the *p* adjacent items. At the end of this step, the root of the heap contains the best such fusion. This is illustrated in Fig. 3 for the RLGH data set. The best candidate fusion, which is by definition the root of the tree, consists in merging $\left\{ 4\right\}$ and $\left\{ 5\right\}$. It is highlighted in violet and the two “neighbor fusions”, *i.e.*, the fusions that involve either $\left\{ 4\right\}$ or $\left\{ 5\right\}$, are highlighted in pink. The initialization step has a $\mathcal {O}(p\log (p))$ time complexity because the complexity of inserting each of the $p-1$ elements in the heap is upper bounded by the maximal depth of the heap, that is, $\log _2(p)$.

**Fig. 4:**
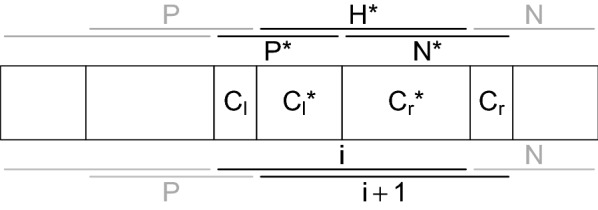
Illustration of the result of a merging step in Algorithm 2

As stated in the previous section, the merging step consists in finding the best candidate fusion (line 5), removing it from the heap (line 6) and inserting (up to) two possible fusions (lines 11–12). The other lines of the algorithm explain how the information regarding the adjacent fusions and clusters are retrieved and updated. The notation is illustrated in Fig. 4, elaborating on the example of Fig. 2.

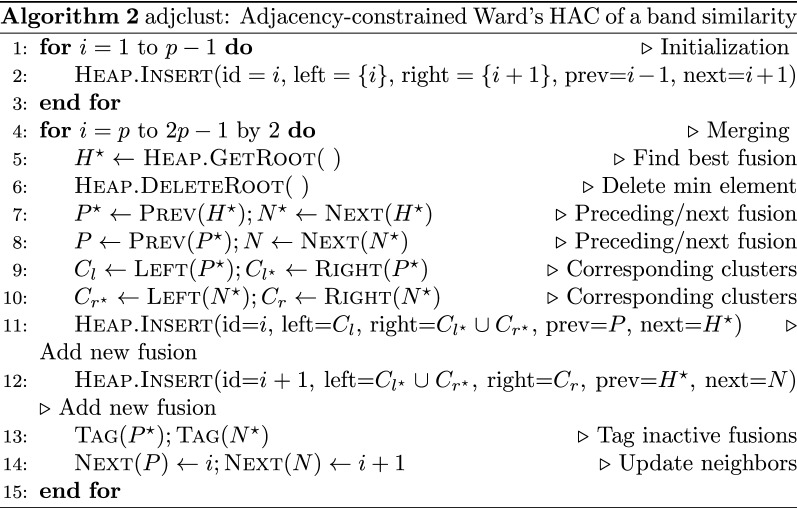



**Fig. 5:**
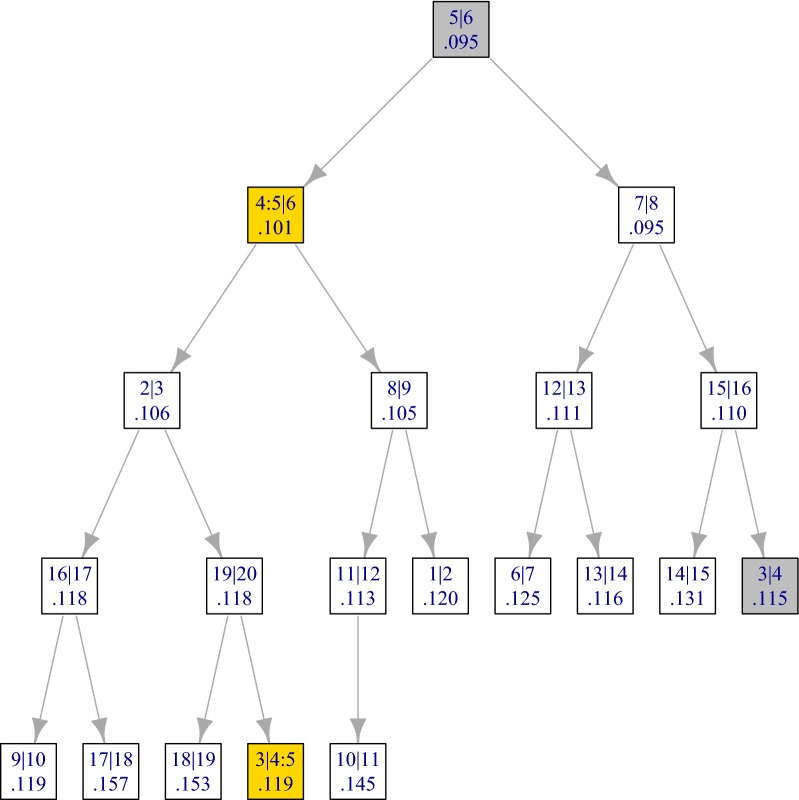
Min heap after the first merging step for the RLGH data set. The nodes corresponding to the fusion that have changed since initialization (Fig. 3) are highlighted

The state of the heap after the first fusion is illustrated by Fig. 5, where the two new candidate fusions are highlighted in yellow. The two fusions highlighted in grey are the neighbors of the first fusion.

In Algorithm 2, we have omitted several points for simplicity and conciseness of exposition. For a more complete description, the following remarks can be made:

1. The calculation of the linkage is not mentioned explicitly in the calls to Heap.Insert. As explained in “Ward’s linkage as a function of pre-calculated sums” section, the linkage between any two clusters can be calculated in constant time from pre-calculated pencil sums.
2. Algorithm 2 should take appropriate care of cases when the best fusion involves the first or last cluster. In particular, only one new fusion is defined and inserted in such cases. This is taken care of in the **adjclust** package, but not in Algorithm 2 for simplicity of exposition.
3. At each merging step the algorithm also tags as inactive the fusions involving the merged clusters (13). Indeed, once a cluster is fused with its left neighbor it can no longer be fused with its right neighbor and vice-versa. These fusions are highlighted in pink in Fig. 3 and in gray (once tagged) in Fig. 5. In order to avoid invalid fusions, each candidate fusion has an active/inactive label (represented by the gray highlight in Fig. 5), and when retrieving the next best candidate fusion (line 5), the min heap is first cleaned by deleting its root as long as it corresponds to an inactive fusion. In the course of the whole algorithm, this additional cleaning step will at worst delete 2*p* roots for a total complexity of $\mathcal {O}(p\log (p))$.
4. The insertion instructions in Algorithm 2 indicate that the heap not only contains the value of the candidate fusions, but also the left and right clusters of each fusion, and the preceding and next candidate fusions in the order of the original objects to be clustered. In practice this side information is not actually stored in the heap, but in a dedicated array, together with the values of the corresponding linkage and the validity statuses of each candidate fusion. The heap only stores the index of each fusion in that array. The state of this array before and after the first fusion for the RLGH data set are given in Tables 2 and 3.

**Table 2:** State of the array after initialization of the clustering for the RLGH data set, as in Fig. 3

Left	Right	Prev	Next	Linkage	Valid
1	2	NA	2	0.121	1
2	3	1	3	0.106	1
3	4	2	4	0.115	1
4	5	3	5	0.095	1
5	6	4	6	0.095	1
$\vdots$		$\vdots$			$\vdots$
18	19	17	19	0.153	1
19	20	18	NA	0.118	1

**Table 3:**
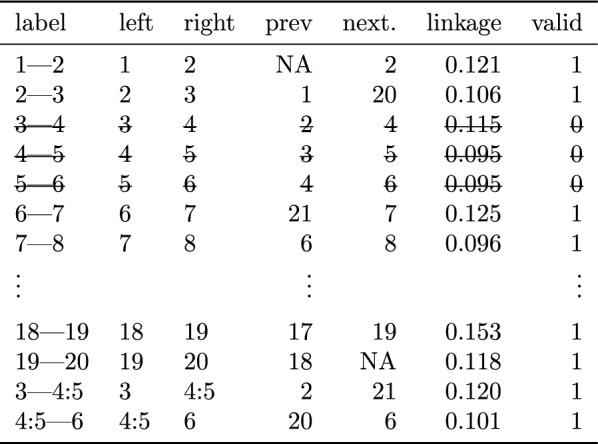
State of the array after the first merge in the clustering for the RLGH data set, as in Fig. 5

#### Complexity of the proposed algorithm

By pre-calculating the *ph* initial pencils recursively using cumulative sums, the time complexity of the pre-computation step is *ph* and the time complexity of the computation of the linkage of the merged cluster with its two neighbors is $\mathcal {O}(1)$ (see Section S1.2 of Additional file 1 for further details). Its total time complexity is thus $\mathcal {O}(p(h + \log (p))$, where $\mathcal {O}(ph)$ comes from the pre-computation of pencils, and $\mathcal {O}(p\log (p))$ comes from the *p* iterations of the algorithm (to merge clusters from *p* clusters up to 1 cluster), each of which has a complexity of $\mathcal {O}(\log (p))$. The space complexity of this algorithm is $\mathcal {O}(ph)$ because the size of the heap is $\mathcal {O}(p)$ and the space complexity of the pencil pre-computations is $\mathcal {O}(ph)$. Therefore, the method achieves a quasi-linear (linearithmic) time complexity and linear space complexity when $h \ll p$, which in our experience is efficient enough for analyzing large genomic datasets.

#### Implementation

Our method is available in the R package **adjclust**, using an underlying implementation in C and available on CRAN.^2^ Additional features have been implemented to make the package easier to use and results easier to interpret. These include:

- Plots to display the similarity or dissimilarity together with the dendrogram and a clustering corresponding to a given level of the hierarchy as illustrated in Additional file 1: Figure S2;
- Wrappers to use the method with SNP data or Hi-C data that take data from standard bed files or outputs of the packages **snpStats** and **HiTC** respectively;
- A function to guide the user towards a relevant cut of the dendrogram (and thus a relevant clustering). In practice the underlying number of clusters is rarely known, and it is important to choose one based on the data. Two methods are proposed in **adjclust**: the first is based on a broken stick model [6] for the dispersion. Starting from the root of the dendrogram, the idea is to iteratively check whether the decrease in within-cluster variance corresponding to the next split can or cannot be explained by a broken stick model and to stop if it can. To the best of our knowledge this broken stick strategy is ad hoc in the sense that it does not have a statistical justification in terms of model selection, estimation of the signal, or consistency. The second method is based on the slope heuristic that is statistically justified in the case of segmentation problems [3, 18], for which HAC provides an approximate solution. This later approach is implemented using the **capushe** package [2], with a penalty shape of $p-1 \atopwithdelims ()K-1$.

Clustering with spatial constraints has many different applications in genomics. The next two sections illustrate the relevance of our adjacency constraint clustering approach in dealing with SNP and Hi-C data. In both cases samples are described by up to a few million variables. All simulations and figures were performed using the R package **adjclust**, version 0.5.7.

## Linkage disequilibrium block inference in GWAS

Genome-Wide Association Studies (GWAS) seek to identify causal genomic variants associated with rare human diseases. The classical statistical approach for detecting these variants is based on univariate hypothesis testing, with healthy individuals being tested against affected individuals at each locus. Given that an individual’s genotype is characterized by millions of SNPs this approach yields a large multiple testing problem. Due to recombination phenomena, the hypotheses corresponding to SNPs that are close to each other along the genome are statistically dependent. A natural way to account for this dependence in the process is to reduce the number of hypotheses to be tested by grouping and aggregating SNPs [11, 20] based on their pairwise Linkage Disequilibrium (LD). In particular, a widely used measure of LD in the context of GWAS is the $r^2$ coefficient, which can be estimated directly from genotypes measured by genotyping array or sequencing data using standard methods [9]. The similarity $S = (r^2_{ij})_{i,j}$ induced by LD can be shown to be a kernel (see Section S1.3 of Additional file 1). Identifying blocks of LD may also be useful to define tag SNPs for subsequent studies, or to characterize the recombination phenomena.

Numerical experiments were performed on a SNP dataset coming from a GWA study on HIV [10] based on 317k Illumina genotyping microarrays. For the evaluation we used five data sets corresponding to five chromosomes that span the typical number of SNPs per chromosome observed on this array ($p = 23,304$ for chromosome 1, $p=20,811$ for chromosome 6, $p=14,644$ for chromosome 11, $p=8,965$ for chromosome 16 and $p=5,436$ for chromosome 21).

For each dataset, we computed the LD using the function ld of **snpStats**, either for all SNP pairs ($h=p$) or with a reduced number of SNP pairs, corresponding to a bandwidth $h \in \{100,\ 200,\ 500,\ 1000,\ 2000,\ 5000,\ 10000,\ 20000\}.$ The packages **rioja** [25] (which requires the full matrix to be given as a dist object^3^) and **adjclust** with sparse matrices of the class dgCMatrix (the default output class of ld) were then used to obtain hierarchical clusterings. All simulations were performed on a 64 bit Debian 4.9 server, with 512G of RAM, 3GHz CPU (192 processing units) and concurrent access. The available RAM was enough to perform the clustering on the full dataset ($h=p$) with **rioja** although we had previously noticed that **rioja** implementation could not handle more than 8000 SNPs on a standard laptop because of memory issues.

### Quality of the band approximation

**Fig. 6:**
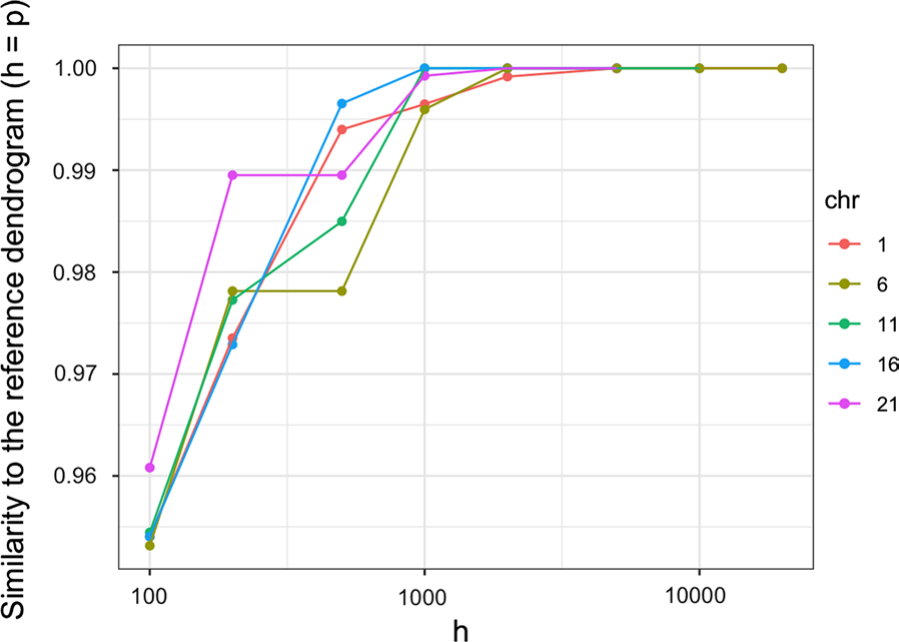
Quality of the band approximation as a function of the bandwidth *h* for five different chromosomes

First, we evaluated the relevance of the band approximation by comparing the dendrogram obtained with $h < p$ to the reference dendrogram obtained with the full bandwidth ($h=p$). To perform this comparison we simply recorded the index *t* of the last clustering step (among $p-1$) for which all the preceding fusions in the two dendrograms are identical. The quantity $t/(p-1)$ can then be interpreted as a measure of similarity between dendrograms, ranging from 0 (the first fusions are different) to 1 (the dendrograms are identical). Figure 6 displays the evolution of $t/(p-1)$ for different values of *h* for the five chromosomes considered here. For example, for all five chromosomes, at $h = 1000$, the dendrograms differ from the reference dendrogram only in the last $0.5\%$ of the clustering step. For $h \ge 2000$ the dendrograms are exactly identical to the reference dendrogram. We also considered other criteria for evaluating the quality of the band approximation, including Baker’s Gamma correlation coefficient [5], which corresponds to the Spearman correlation between the ranks of fusion between all pairs of objects. The results obtained with these indices are not shown here because they were consistent with those reported in Fig. 6.

One important conclusion that may be drawn from these results is that the influence of the bandwidth parameter is the same across chromosomes, that is, across values of *p* (that range from 5000 to 23000 in this experiment). Therefore, it makes sense to assume that *h* does not depend on *p* and that the time and space complexity of our proposed algorithm, which depends on *h*, is indeed quasi-linear in *p*.

### Scalability and computation times

**Fig. 7:**
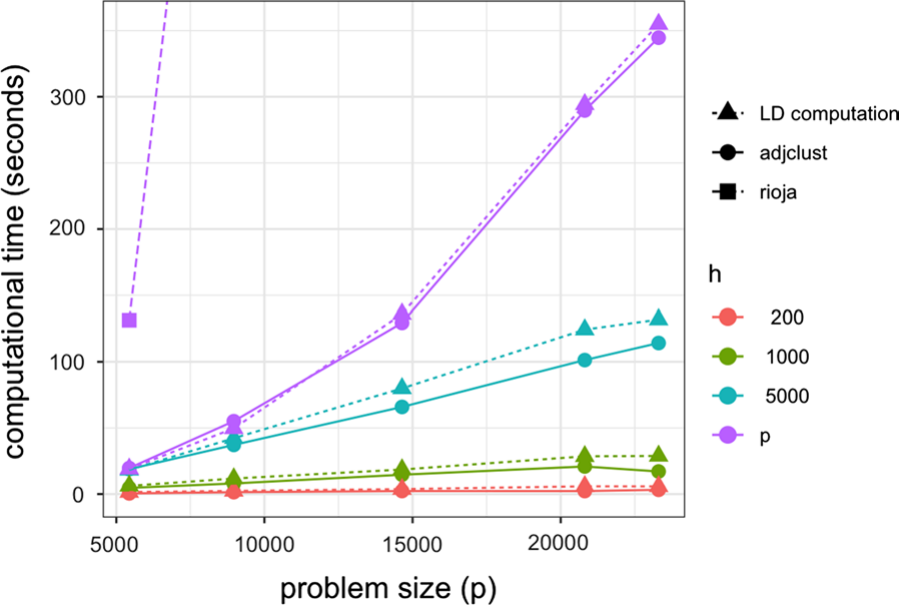
Computation times versus *p*: LD matrices, for CHAC **rioja** and **adjclust** with varying values for the band *h*

Figure 7 displays the computation time for the LD matrix (dotted lines) and for the CHAC with respect to the size of the chromosome (*x* axis), both for **rioja** (dashed line) and **adjclust** (solid lines). As expected, the computation time for **rioja** did not depend on the bandwidth *h*, so we only represented $h=p$. For **adjclust**, the results for varying bandwidths are represented by different colors. Only the bandwidths 200, 1000, and 5000 are representend in Fig. 7 for clarity.

Several comments can be made from Fig. 7. First, the computation times of **rioja** are much larger than those of **adjclust**, even when $h=p$ where both methods implement the exact same algorithm. For the largest chromosome considered here (chromosome 1, $p=23304$), the running time of **rioja** is 18900 seconds (more than 5 h), compared to 345 seconds (less than 6 minutes). As expected, the complexity of **adjclust** with $h=p$ is quadratic in *p*, while it is essentially linear in *p* for fixed values of $h<p$. For large values of *p* the gain of the band approximation is substantial: for $p=23304$ (chromosome 1), the running time of **adjclust** for $h=1000$ (which is a relevant value in this application according to the results of the preceding section) is of the order of 20 s.

We also note that regardless of the value of *h*, the total time needed for the clustering is of the order of (and generally lower than) the time needed for the computation of the LD.

## Hi-C analysis

Hi-C protocol identifies genomic loci that are located nearby in vivo. These spatial co-locations include intra-chromosomal and inter-chromosomal interactions. After bioinformatics processing (alignment, filtering, quality control...), the data are provided as a sparse square matrix with entries that give the number of reads (contacts) between any given pair of genomic locus bins at genome scale. Typical sizes of bins are $\sim$40 kb, which results in more than 75,000 bins for the human genome. Constrained clustering or segmentation of intra-chromosomal maps is a tool frequently used to search for *e.g.*, functional domains (called TADs, Topologically Associating Domains). A number of methods have been proposed for TAD calling (see Forcato et al. [15] for a review and comparison), among which the ones proposed by Fraser et al. [16], Haddad et al. [21] that take advantage of a hierarchical clustering, even using a constrained version for the second reference. In the first article, the authors proceed in two steps with a segmentation of the data into TADs using a Hidden Markov Model on the directionality index of Dixon, followed by a greedy clustering on these TADs, using the mean interaction as a similarity measure between TADs. Proceeding in two steps reduces the time required for the clustering, which is $O(p^2)$ otherwise. However, from a statistical and modeling perspective these two steps would appear redundant. Also, pipelining different procedures (each of them with their sets of parameters) makes it very difficult to control errors. Haddad et al. [21] directly use adjacency-constrained HAC, with a specific linkage that is not equivalent to Ward’s. They do not optimize the computational time of the whole hierarchy, instead stopping the HAC when a measure of homogeneity of the cluster created by the last merge falls below a parameter. Both articles thus highlight the relevance of HAC for exploratory analysis of Hi-C data. Our proposed approach provides, in addition, a faster way to obtain an interpretable solution, using the interaction counts as a similarity and a *h* similar to the bandwidth of the Dixon index.

### Data and method

Data used to illustrate the usefulness of constrained hierarchical clustering for Hi-C data came from Dixon et al. [12], Shen et al. [33]. Hi-C contact maps from experiments in mouse embryonic stem cells (mESC), human ESC (hESC), mouse cortex (mCortex) and human IMR90 Fibroblast (hIMR90) were downloaded from the authors’ website at http://chromosome.sdsc.edu/mouse/hi-c/download.html (raw sequence data are published on the GEO website, accession number GSE35156.

Even if these data do not perfectly fulfill the sparse band assumption, their sparsity is very high, especially outside a band centered on the diagonal. Taking as an example the largest and smallest chromosomes of the hESC data (chromosomes 1 and 22 respectively), the proportion of bin pairs with a positive count (*present* bin pairs) correspond to 10.7% and 25.8% respectively. This proportion is even smaller when focusing on bins pairs with a count larger than one (3.2% and 10.5% respectively). In addition, these bin pairs are mostly concentrated close to the diagonal: the proportion of present bin pairs that are located within a 10% diagonal band correspond to 60.1% and 45.6% of the present bin pairs, respectively. Finally, respectively 92.5% and 87.8% of the remaining present bin pairs have a count equal to only 1.

All chromosomes were processed similarly:

- Counts were $\log$-transformed to reduce the distribution skewness;
- Constrained hierarchical clustering was computed on $\log$-transformed data using, for the similarity, either the whole matrix ($h=p$) or the sparse approach with a sparse band size equal to $h = \{0.5p, 0.1p\}$;
- Model selection was finally performed using both the broken stick heuristic and the slope heuristic.

All computations were performed using the Genotoul cluster.

### Influence of the bandwidth parameter

**Fig. 8:**
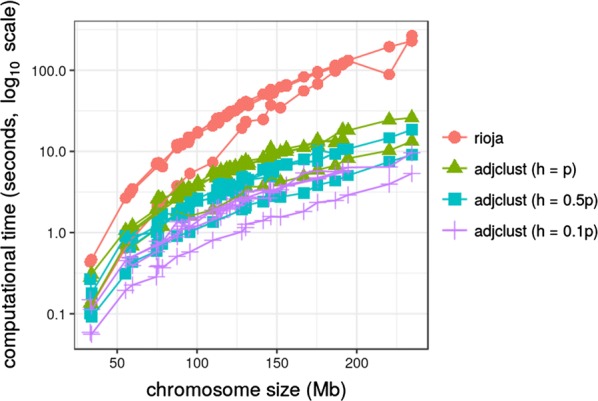
Impact of sparsity on the computational time. Dots that correspond to the same datasets but different chromosomes are linked by a path

The effect of *h* (sparse band parameter) on computational time, dendrogram organization and clustering were assessed. Figure 8 gives the computational times versus the chromosome size for the three values of *h* together with the computational time obtained by the standard version of constrained hierarchical clustering as implemented in the R package **rioja**. As expected, the computational time is substantially reduced by the sparse version (even though not linearly with respect to *h* because of the preprocessing step that extracts the band around the diagonal), making the method suitable for dealing efficiently with a large number of chromosomes and/or a large number of Hi-C experiments. **rioja**, that cannot cope efficiently with the sparse band assumption, requires considerably more computational time (10 times the time needed by **adjclust**). In addition, the memory required by the two approaches is very different: **adjclust** supports sparse matrix representation (as implemented in the R package **Matrix**), which fits the way Hi-C matrices are typically stored (usually these matrices are given as rows with bin number pairs and associated count). For instance, the sparse version (dsCMatrix class) of the largest chromosome (chromosome 1) in the hESC data is 23 Mb, as opposed to 231 Mb for the full version. The sparse version of the smallest chromosome (chromosome 22) is 1.1 Mb, versus 5.2 Mb for the full version. The sparse version of the $h=0.1p$ band for these two chromosomes is, respectively, 13.2 M and 0.4 Mb respectively.

However, this gain in time and space did not impact the results of the method: the indexes of the first difference were computed between the dendrograms obtained by the full version ($h=p$) and by the two sparse versions ($h \in \{0.5p, 0.1p\}$) for every chromosome. For most of the clusterings there was no difference in merge for $h=0.5p$ (with the similarity computed as in Fig. 6 always larger than 0.9992, and equal to 1 in more than 3 clusterings out of 4). For $h=0.1p$, the similarity ranged from 0.9811 to 0.9983. Baker’s Gamma index and Rand indices [24] for selected clusterings (both with broken stick and slope heuristic) confirmed this conclusion (results not shown).

### Results

Additional file 1: Figure S1 provides the average cluster size for each chromosome versus the chromosome length. It shows that the average cluster size is fairly constant among the chromosomes and does not depend on the chromosome length. Both model selection methods found typical cluster sizes of 1-2 Mb, which is in line with what is reported in Forcato et al. [15] for some TAD callers.

Additional file 1: Figure S2 shows that clusters for a given chromosome (here chromosome 11 for hIMR90 and chromosome 12 for mCortex) can have different sizes and also different interpretations: some clusters exhibit a dense interaction counts (deep yellow) and are thus good TAD candidates whereas a cluster approximately located between bin 281 and bin 561 in chr12-mCortex map has almost no interaction and can be viewed as possibly separating two dense interaction regions.

**Fig. 9:**
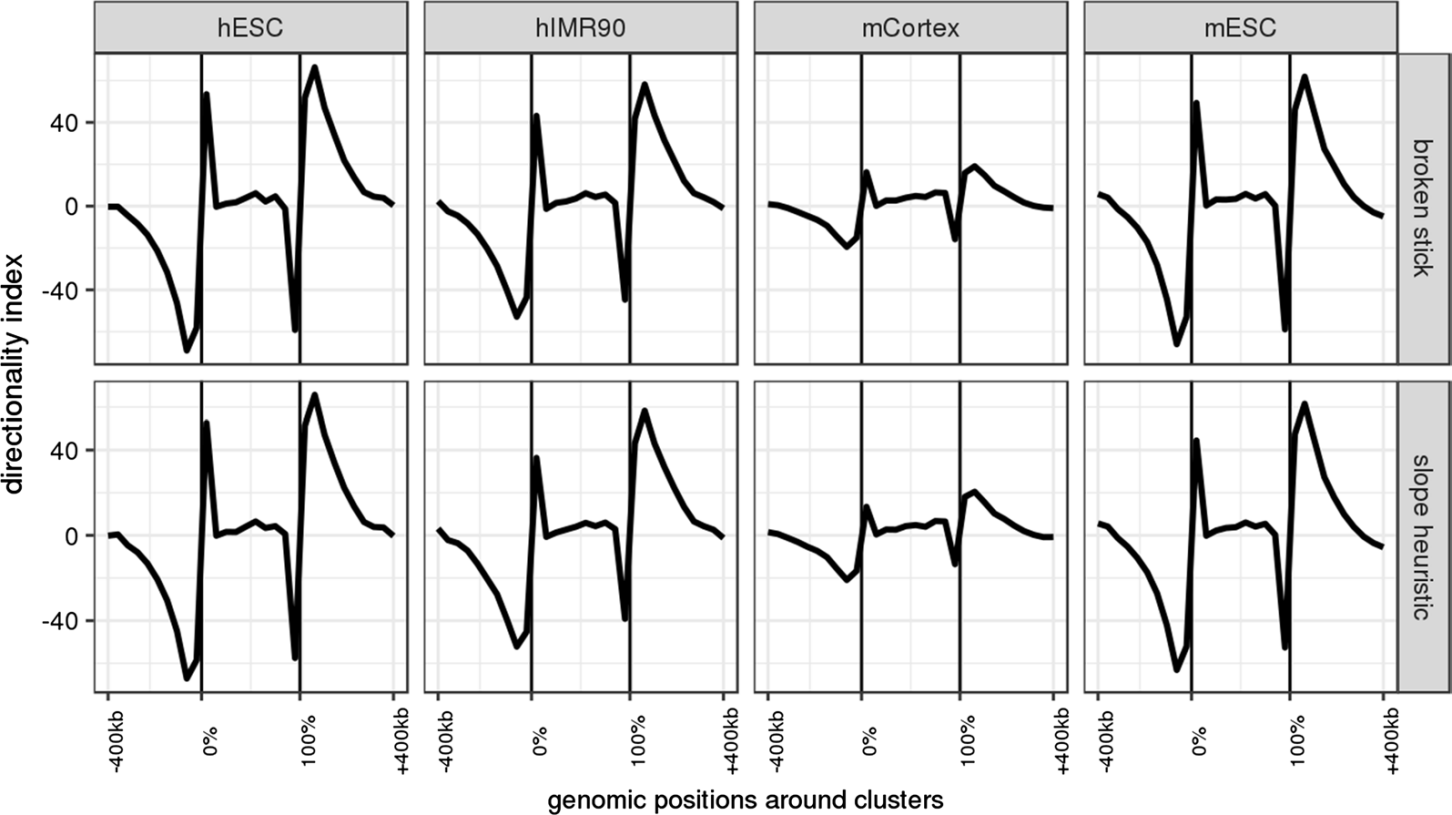
Evolution of the Directionality Index (DI) around clusters

The directionality Index (DI, Dixon et al. [12]) quantifies a directional (upstream vs downstream) bias in interaction frequencies, based on a $\chi ^2$ statistic. DI is the original method used for TAD calling in Hi-C. Its sign is expected to change and DI values are expected to show a sharp increase at TADs boundaries. Figure 9 displays the average DI, with respect to the relative bin position within the cluster and the absolute bin position outside the cluster. The clusters found by constrained HAC show a relation with DI that is similar to what is expected for standard TADs, with slightly varying intensities.

Finally, boundaries of TADs are known to be enriched for the insulator binding protein CTCF Dixon et al. [12]. CTCF ChIP-seq peaks were retrieved from ENCODE [13] and the distribution of the number of the 20% most intense peaks was computed at $\pm 400$ Kb of cluster boundaries, as obtained with the broken stick heuristic (Additional file 1: Figure S3). The distribution also exhibited an enrichment at cluster boundaries, which indicates that the clustering is relevant with respect to the functional structure of the chromatin.

## Conclusions

We have proposed an efficient approach to perform constrained hierarchical clustering based on kernel (or similarity) datasets with several illustrations of its usefulness for genomic applications. The method is implemented in a package that is shown to be fast and that currently includes wrappers for genotyping and Hi-C datasets. The package also provides two possible model selection procedures to choose a relevant clustering in the hierarchy. The output of the method is a dendrogram, which can be represented graphically, and provides a natural hierarchical model for the organization of the objects.

The only tuning parameter in our algorithm is the bandwidth *h*. The numerical experiments reported in this paper suggest that at least for GWAS and Hi-C studies, there exists a range of values for *h* such that $h \ll p$ (which implies very fast clustering) and the result of the HAC is identical or extremely close to the clustering obtained for $h=p$. While the range of relevant values of *h* will depend on the particular application, an interesting extension of the present work would be to propose a data-driven choice of *h* by running the algorithm on increasing (yet small) values for *h* on a single chromosome, and deciding to stop when the dendrogram is stable enough. In addition, by construction, all groups smaller than *h* are identical in both clusterings (with and without the *h*-band approximation).

While HAC is a tool for *exploratory* data analysis, an important prospect of the present work will be to make use of the low time and memory footprint of the algorithm in order to perform *inference* on the estimated hierarchy using stability/resampling-based methods. Such methods could be used to propose alternative model selection procedures, or to compare hierarchies corresponding to different biological conditions, which has been shown to be relevant to Hi-C studies [16].

## Supplementary information

**Additional file 1.** Supplementary methods and results.

## Data Availability

GWAS data analyzed in this paper are available as described in “Linkage disequilibrium block inference in GWAS” section. Hi-C data analyzed in this paper are available as described in “Data and method” section.

## Footnotes

1 Available on CRAN at https://cran.r-project.org/package=rioja.

2 https://cran.r-project.org/package=adjclust.

3 The time needed to compute this matrix was 50-1000 times larger than the computation of the LD matrix itself. However, we did not include this in the total computation time required by **rioja** because we have not tried to optimize it from a computational point of view.
