# Supplementary material for: Adjacency-constrained hierarchical clustering of a band similarity matrix with application to genomics
Source: Algorithms Mol Biol. 2019 Nov 15;14:22. doi: 10.1186/s13015-019-0157-4 (PMC6857244; doi:10.1186/s13015-019-0157-4)
Supplement: Supplementary file 1 — Additional file 1. Supplementary methods and results. [file 13015_2019_157_MOESM1_ESM.pdf]

Additional file for "Adjacency-constrained hierarchical clustering of a band similarity matrix with application to Genomics"

Christophe Ambroise[1], Alia Dehman[2], Pierre Neuvial[3],
Guillem Rigaill[1,4] and Nathalie Vialaneix[5]

[1] Laboratoire de Mathématiques et Modélisation d'Evry, UMR CNRS 8071, Université d'Evry Val d'Essonne, 23 boulevard de France, 91037 Evry, France.
christophe.ambroise@univ-evry.fr
[2] Hyphen-stat, 195 Route d'Espagne, 31036 Toulouse, France.
alia.dehman@hyphen-stat.com
[3] Institut de Mathématiques de Toulouse, UMR5219 CNRS, Université de Toulouse, UPS IMT, F-31062 Toulouse Cedex 9, France.
pierre.neuvial@math.univ-toulouse.fr
[4] Institute of Plant Sciences Paris Saclay IPS2, CNRS, INRA, Gif sur Yvette, France.
guillem.rigaill@inra.fr
[5] MIAT, Université de Toulouse, INRA, Castanet-Tolosan, France.
nathalie.vialaneix@inra.fr

Contents

S1 Supplementary methods **1**
S1.1 Proof of Equation (1) . . . 1
S1.2 Time and space complexity of the pencil trick . . . 3
S1.3 Linkage disequilibrium and kernel . . . 3

S2 Supplementary results **5**

S1 Supplementary methods

S1.1 Proof of Equation (1)

Proof of Equation (1). The theory of Reproducing Kernel Hilbert Spaces [Aronszajn, 1950] makes is possible to generalize the definition of Ward-based HAC to the case where the similarity matrix S describing the objects to cluster is a kernel, *i.e.*, a positive definite symmetric matrix. In this case, there exists a unique Hilbert space $(\mathcal{H}, \langle ., . \rangle_{\mathcal{H}})$ and a feature map $\phi : \mathcal{X} \rightarrow \mathcal{H}$, where $\mathcal{X}$ denotes the arbitrary set in which the objects, $\{x_1, \ldots, x_p\}$, described by s are defined, such that the kernel s corresponds to the dot product in $\mathcal{H}$:

$$s_{ij} = \langle \phi(x_i), \phi(x_j) \rangle_{\mathcal{H}}. \tag{S1}$$

Following Murtagh and Legendre [2014], since the feature space $\mathcal{H}$ is Euclidean, Ward's linkage may be written as

$$\forall C,\, C' \subset \{x_1, \ldots, x_p\},\; C \cap C' = \varnothing, \qquad \delta(C, C') = \frac{|C||C'|}{|C| + |C'|} \|\bar{C} - \bar{C}'\|^2_{\mathcal{H}}$$

where for any cluster C, $\bar{C} := \frac{1}{|C|} \sum_{i \in C} \phi(x_i)$ is the center of gravity of C in $\mathcal{H}$ and $\| \cdot \|^2_{\mathcal{H}}$ is the norm associated to the scalar product in $\mathcal{H}$. However,

$$\begin{aligned} \|\bar{C} - \bar{C}'\|^2_{\mathcal{H}} &= \langle \bar{C} - \bar{C}', \bar{C} - \bar{C}' \rangle_{\mathcal{H}} \\ &= \langle \bar{C}, \bar{C} \rangle_{\mathcal{H}} + \langle \bar{C}', \bar{C}' \rangle_{\mathcal{H}} - 2 \langle \bar{C}, \bar{C}' \rangle_{\mathcal{H}} \,. \end{aligned}$$

Then, Equation (S1) yields $\forall C,\, C' \subset \{x_1, \ldots, x_p\}$,

$$\langle \bar{C}, \bar{C}' \rangle_{\mathcal{H}} = \frac{1}{|C||C'|} \langle \sum_{i \in C} \phi(x_i), \sum_{i \in C'} \phi(x_i) \rangle_{\mathcal{H}} = \frac{1}{|C||C'|} S_{CC'} \,.$$

where $S_{CC'} = \sum_{i \in C, j \in C'} s_{ij}$. This implies

$$\delta(C, C') = \frac{|C||C'|}{|C| + |C'|} \left(\frac{S_{CC}}{|C|^2} + \frac{S_{C'C'}}{|C'|^2} - \frac{2 S_{CC'}}{|C||C'|} \right)$$

where $S_{CC} = S(C)$ and $S_{C'C'} = S(C')$. To conclude, we note that

$$\begin{aligned} S(C \cup C') &= S(C) + S(C') + 2 \sum_{i \in C, j \in C'} s_{ij} \\ &= S_{CC} + S_{C'C'} + 2 S_{CC'} \,, \end{aligned}$$

so that

$$\begin{aligned} \delta(C, C') &= \frac{|C||C'|}{|C| + |C'|} \left(\left(\frac{1}{|C|^2} + \frac{1}{|C||C'|} \right) S(C) + \left(\frac{1}{|C'|^2} + \frac{1}{|C||C'|} \right) S(C') - \frac{1}{|C||C'|} S(C \cup C') \right) \\ &= \frac{S(C)}{|C|} + \frac{S(C')}{|C'|} - \frac{S(C \cup C')}{|C \cup C'|} \end{aligned}$$

which concludes the proof. □

Because $\delta(C, C')$ is explicitly written in terms of S only, Ward's HAC can be performed implicitly in the feature space, without an explicit calculation. In particular, the mapping ϕ itself does not need to be known explicitly. This property is known as the kernel trick. In our paper, the kernel trick is used to write the distance between the centers of gravity in the feature space as a function of S only. Ward's HAC is then obtained by iteratively updating the matrix of similarities between all pairs of centers of gravity after each successive merge. To the best of our knowledge, the formulation of Ward's linkage in terms of sums of elements of the similarity matrix S has never been explicitly written in the form of Equation (1) even if kernel-based HAC has already been proposed by Qin et al. [2003], Ah-Pine and Wang [2016] for linkages including Ward's linkage, but

S1.2 Time and space complexity of the pencil trick

Space complexity The pencil trick is based on the pre-computation of backward and forward pencils as defined in Equation (2). By construction, the number of all the bandwidths of the pencils involved is less than h. Therefore, only pencils $P(r, l)$ and $\bar{P}(r, l)$ with $1 \leq r \leq p$ and $1 \leq l \leq h$ have to be pre-computed and the total number of pencils to compute and stored is less than $2ph$. The space complexity is thus $\mathcal{O}(ph)$ for pencils.

Time complexity The contribution of the pencil trick to the algorithm complexity is divided into:

- during the initialization of the algorithm, the **pre-computation of the backward and forward pencils**. This can be done efficiently by a recursive computation, as described in Algorithm S1. This algorithm is based on the computation of less than ph quantities, all having a complexity equal to 1. The total time complexity of the method is thus $\mathcal{O}(ph)$;

Algorithm S1 Pencil trick: Precomputation of pencils by a recursive strategy

for $i = 1$ to p **do**
    $P(i, 1) \leftarrow s(i, i)$ ▷ $\mathcal{O}(1)$ for every i
    for $l = 2$ to $\min(h, p + 1 - i)$ **do**
        $P(i, l) \leftarrow P(i, l - 1) + s(i, i + l - 1)$ ▷ $\mathcal{O}(1)$ for every i and every l
    end for
end for

- during the call to Heap.Insert (see Section 2.2.2 and Algorithm 2 of the main text), the computation of the linkage values between the new(ly fused) cluster and its right and left neighbors. The time complexity of each linkage computation is $\mathcal{O}(1)$ (constant) because according to Equations (1) and (4), δ is a function of a constant number of pencils.

S1.3 Linkage disequilibrium and kernel

If SNP values at position i are modeled by a binary random variable Z_i which is the indicator of the presence of minor allele for this locus, it is standard to make the asumption that

$$Z_i \sim \mathcal{B}(p_i).$$

The linkage disequilibrium (LD) between locus i and locus j is defined as the covariance between the two corresponding random variables:

$$D_{ij} = p_{ij} - p_i p_j = E[Z_i Z_j] - p_i p_j = \mathrm{Cov}(Z_i, Z_j).$$

For practical use the measure is normalized to be between zero and one. Two popular choices of normalization are the squared correlation

$$r^2(i, j) = \mathrm{Cor}(Z_i, Z_j)^2$$

and

$$d'_{ij}(i, j) = \frac{D(i, j)}{\max_{ij} D(i, j)}.$$

In this paper, we consider the r^2 which is a classical choice in the context of association studies. More precisely, given observations of the genotypes, or n individuals, we denote by $\boldsymbol{z}_i$ the $2n$-dimensional vector of normalized allele values of locus i for the $2n$ genotypes of the n individuals and estimates the LD with $k(i,j) := \left(\sum_{\ell=1}^{2n} \boldsymbol{z}_{i\ell}\boldsymbol{z}_{j\ell}\right)^2$.

It is possible to prove that this estimation, k, is a kernel, *i.e.*, a positive definite symmetric matrix. This result comes from the fact that $k(i,j)$ can be re-written as:

$$\sum_{\ell,\ell'=1}^{2n} (z_{i\ell}z_{j\ell})(z_{i\ell'}z_{j\ell'}).$$

Defining the mapping Φ from $\mathbb{R}^{(2n)}$ to $\mathbb{R}^{(2n)^2}$ such that

$$\forall(\ell,\ell') \in \{1,\ldots,2n\}^2, \qquad \Phi(\boldsymbol{z})_{2n(\ell-1)+\ell'} = z_\ell z_{\ell'},$$

we have:

$$k(i,j) = \langle \boldsymbol{z}_i, \boldsymbol{z}_j \rangle^2 = \langle \Phi(\boldsymbol{z}_i), \Phi(\boldsymbol{z}_j) \rangle,$$

which concludes the proof since k is expressed as a dot product with the feature map $i \to \Phi(\boldsymbol{z}_i)$.

Notice that, when the available data are SNPs, computing the square correlation between of two loci raises the additional problem of unknown haplotype phase. Indeed, with association study, we observe locus values for pairs of chromosomes and not for specific chromosomes. For each locus, SNP data give access to a 3×3 contingency table of genotype counts for each pair of loci, while we would like to have the 2×2 table of diplotype counts. Nevertheless, these are classical approaches for estimating the diplotypes from the genotypes.

S2 Supplementary results

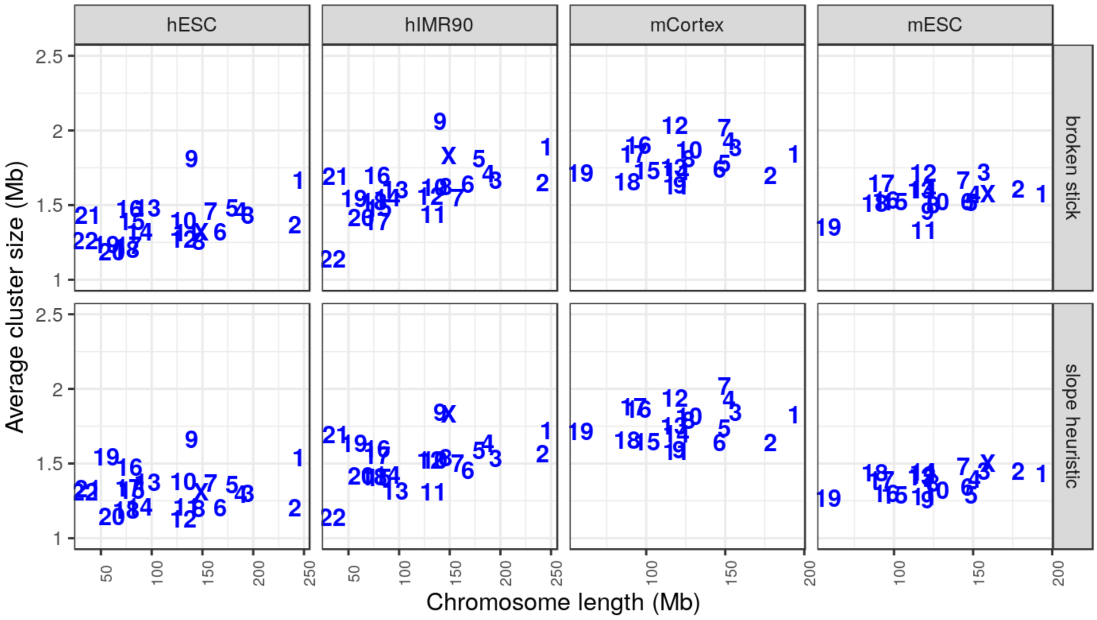

Figure S1: Average cluster size for both model selection approaches, compared to the chromosome length (in term of number of observed bins) for every chromosome and every experiment (full version). Chromosome X in mCortex had an average cluster size larger than 2.5Mb and was thus excluded from the picture.

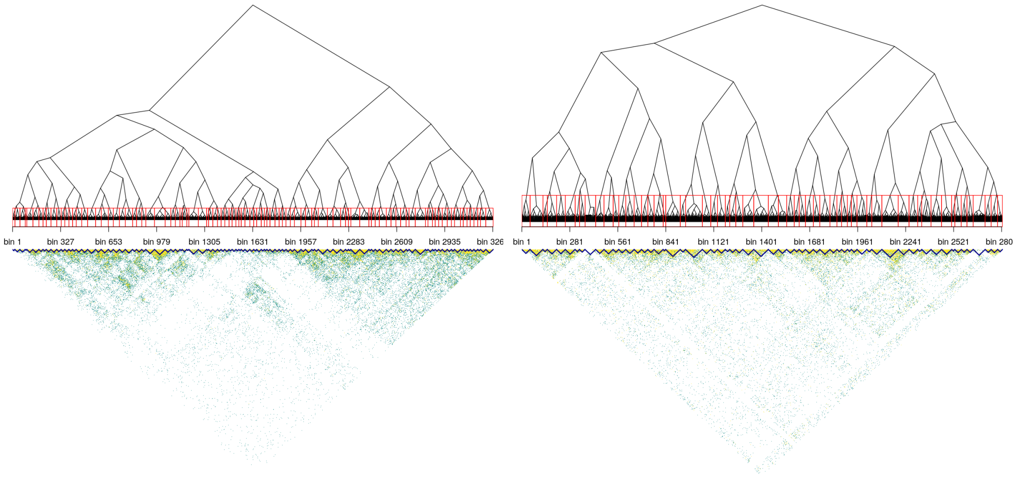

Figure S2: Left: Chromosome 11 of hIMR90. Right: Chromosome 12 of mCortex. Bottom: Hi-C data (log-scaled) with clustering selected by the slope heuristic (blue line). Top: Constrained hierarchical clustering with clustering selected by the slope heuristic (red rectangles).

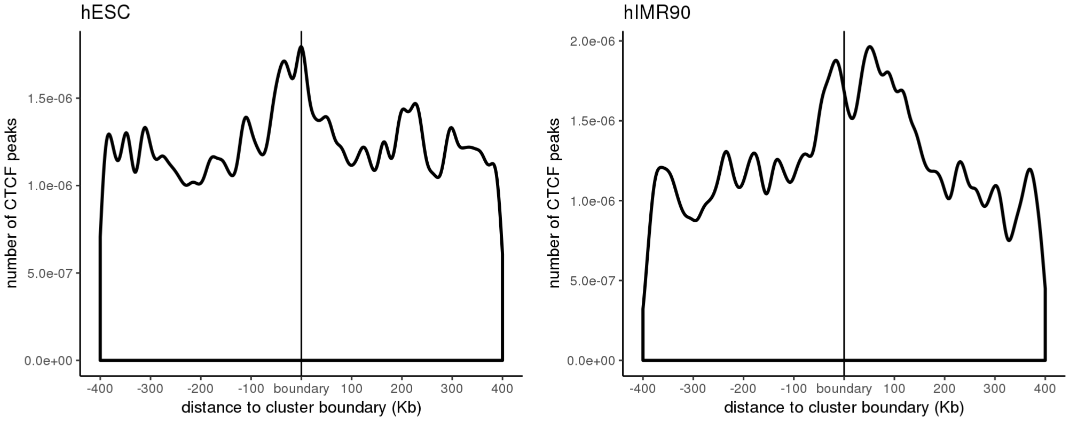

Figure S3: Distribution of the number of the 20% most intense CTCF ChIP-seq peaks with respect to distance of cluster boundaries, as obtained with the broken stick heuristic. Left: hESC. Right: hIMR90.

References

J. Ah-Pine and X. Wang. Similarity based hierarchical clustering with an application to text collections. In H. Boström, A. Knobbe, C. Soares, and P. Papapetrou, editors, *Proceedings of the 15th International Symposium on Intelligent Data Analysis (IDA 2016)*, Lecture Notes in Computer Sciences, pages 320–331, Stockholm, Sweden, 2016. doi: 10.1007/978-3-319-46349-0. URL https://hal.archives-ouvertes.fr/hal-01437124.

N. Aronszajn. Theory of reproducing kernels. *Transactions of the American Mathematical Society*, 68(3):337–404, 1950.

F. Murtagh and P. Legendre. Ward's hierarchical agglomerative clustering method: which algorithms implement ward's criterion. *Journal of Classification*, 31:274–295, 2014. doi: 10.1007/s00357-014-9161-z.

J. Qin, D. P. Lewis, and W. S. Noble. Kernel hierarchical gene clustering from microarray expression data. *Bioinformatics*, 19(16):2097–2104, 2003. doi: 10.1093/bioinformatics/btg288.
